# Predicting ADL disability in community-dwelling elderly people using physical frailty indicators: a systematic review

**DOI:** 10.1186/1471-2318-11-33

**Published:** 2011-07-01

**Authors:** Joan Vermeulen, Jacques CL Neyens, Erik van Rossum, Marieke D Spreeuwenberg, Luc P de Witte

**Affiliations:** 1 School for Public Health and Primary Care (CAPHRI), Maastricht University, Maastricht, The Netherlands; 2 Research Centre Technology in Care, Zuyd University of Applied Sciences, Heerlen, The Netherlands

## Abstract

**Background:**

Disability in Activities of Daily Living (ADL) is an adverse outcome of frailty that places a burden on frail elderly people, care providers and the care system. Knowing which physical frailty indicators predict ADL disability is useful in identifying elderly people who might benefit from an intervention that prevents disability or increases functioning in daily life. The objective of this study was to systematically review the literature on the predictive value of physical frailty indicators on ADL disability in community-dwelling elderly people.

**Methods:**

A systematic search was performed in 3 databases (PubMed, CINAHL, EMBASE) from January 1975 until April 2010. Prospective, longitudinal studies that assessed the predictive value of individual physical frailty indicators on ADL disability in community-dwelling elderly people aged 65 years and older were eligible for inclusion. Articles were reviewed by two independent reviewers who also assessed the quality of the included studies.

**Results:**

After initial screening of 3081 titles, 360 abstracts were scrutinized, leaving 64 full text articles for final review. Eventually, 28 studies were included in the review. The methodological quality of these studies was rated by both reviewers on a scale from 0 to 27. All included studies were of high quality with a mean quality score of 22.5 (SD 1.6). Findings indicated that individual physical frailty indicators, such as weight loss, gait speed, grip strength, physical activity, balance, and lower extremity function are predictors of future ADL disability in community-dwelling elderly people.

**Conclusions:**

This review shows that physical frailty indicators can predict ADL disability in community-dwelling elderly people. Slow gait speed and low physical activity/exercise seem to be the most powerful predictors followed by weight loss, lower extremity function, balance, muscle strength, and other indicators. These findings should be interpreted with caution because the data of the different studies could not be pooled due to large variations in operationalization of the indicators and ADL disability across the included studies. Nevertheless, our study suggests that monitoring physical frailty indicators in community-dwelling elderly people might be useful to identify elderly people who could benefit from disability prevention programs.

## Background

In ageing Western societies, the prevalence of frailty and its adverse outcomes increases [1]. Disability in Activities of Daily Living (ADL), which are the essential activities that a person needs to perform to be able to live independently [2], is an adverse outcome of frailty that places a high burden on frail individuals, care professionals and health care systems [3]. Frail elderly people have a higher risk of ADL disability compared to non-frail elderly people [4-6]. Effective interventions that prevent disability can diminish the burden caused by frailty. For the development of such interventions and the identification of people who might benefit from them, it is important to know which factors predict frailty-related ADL disability.

Frailty is a concept that has been defined in many different ways [7-9]. Various physical, cognitive, psychological, nutritional and social factors have been claimed to contribute to frailty [10]. A definition of frailty that is often used by geriatricians is the following: 'a biologic syndrome of decreased reserve and resistance to stressors, resulting from cumulative decline across multiple physiologic systems, and causing vulnerability to adverse outcomes' [11]. The well known frailty phenotype by Fried et al. [12] which classifies people into categories of robust, pre-frail or frail fits within this physiologic approach of frailty. The frailty phenotype postulates that five indicators of physical functioning (unintentional weight loss, exhaustion, slow walking speed, low grip strength, and low physical activity) are related to each other in a cycle of frailty. A person with none of the indicators is robust, a person with 1 or 2 indicators is pre-frail, and a person with 3 or more indicators is frail. Elderly people who are frail according to the phenotype have a higher risk of disability [4-6].

Although evidence exists that the phenotype predicts disability, it always involves a combination of the five indicators and provides no insight into the predictive value of the individual indicators. Besides that, the phenotype does not provide insight into the predictive value of other possible indicators of physical functioning that might relate to frailty. If individual indicators can predict ADL disability this could be clinically useful in identifying elderly people who might benefit from an intervention that prevents disability or increases physical functioning in daily life. A systematic literature review was conducted to investigate this in community-dwelling elderly people.

## Methods

### Search strategy

Potentially relevant articles were obtained by performing a search in three databases (PubMed, CINAHL, and EMBASE) from January 1975 until April 2010. This cut-off point was chosen because the term frailty was first introduced around the 1980's. To specify the study population the MESH term "aged" was combined with terms such as "frail*", "vulnerable", "low functioning", or "community-dwelling" where * denotes truncated terms. To specify the physical frailty indicators terms such as "grip strength", "weight loss", "balance", "exhaustion", "walking speed", "gait", "physical activity", and related MESH terms were combined with OR. To specify the outcome measure terms such as "disabil*", "Activities of daily living", "functional decline", and related MESH terms were combined with OR. To specify the study design terms such as "cohort studies", "longitudinal", "prognos*", "predict*", and related MESH terms were combined with OR. The searches for study population, physical frailty indicators, outcome, and study design were combined with AND, resulting in the final search. Reference lists of selected reviews and studies were screened for relevant publications that were not identified in the original search. Relevant studies found in these reference lists that met all inclusion criteria were also included in the review.

### Study selection

Articles were eligible for inclusion if they met the following inclusion criteria: 1) written in English or Dutch, 2) a prospective longitudinal design, 3) involving community-dwelling elderly people aged 65 years or older, 4) at least 1 physical frailty indicator as independent variable, and 5) ADL disability as outcome measure. Most recent studies on disability in elderly persons focus on the ability or difficulty in carrying out ADL [13]. The fact that people who suffer from ADL disability, cannot live independently justifies the use of this measure as a key outcome [2]. Articles with only mobility disability as outcome variable were not included because this does not reflect the much broader concept of ADL disability. Studies that only focused on elderly patients with a disease such as Parkinson, depression, or stroke were excluded from the review.

All retrieved articles were first reviewed by two independent reviewers (JV & JCLN) based on their title. In case of disagreement or doubt, the article was included in the second phase of the selection process where all abstracts were assessed. Both reviewers independently labeled the remaining abstracts as 'include' or 'exclude'. Disagreement was resolved by consensus and if consensus could not be reached a third reviewer was consulted (MDS). In the third phase of the selection process, the full-text of the articles was retrieved and reviewed by both reviewers independently. Disagreement was resolved by consensus. In two cases the third reviewer had to be consulted. Agreement between the two independent reviewers in the second and third phase of the selection process was checked by calculating Cohen's Kappa.

### Quality assessment & Data extraction

The quality of the included articles was assessed by both reviewers independently using a list of 27 criteria (see Table 1). This list was constructed based on previous research on methodological quality, quality of reporting criteria for observational research, and previous reviews regarding prediction of disability [14-17]. Each item was scored with 0 or 1 resulting in a possible range of 0 to 27 points per included study. A higher score indicated higher quality.

**Table 1:** List of quality criteria

**Nr**.	Criteria	Yes = 1	No = 0
1	Was the rationale of the research described?		

2	Were the objectives of the research clearly stated?		

3	Was the study a prospective cohort study?		

4	Was the follow-up of the cohort study 5 years or longer?		

5	Were the key-elements of the study design described?		

6	Were the setting, relevant dates and timeframe of the research described?		

7	Were the eligibility criteria for participants described?		

8	Were the participants free of disability at baseline?		

9	Were the predictors and dependent variables described?		

10	Were the measurement methods for the predictors and dependent variables described?		

11	Were standardized or valid measurements used for the predictors?		

12	Were standardized or valid measurements used for the outcome?		

13	Were potential types of bias addressed?		

14	Was it clear how the quantitative data were handled in the analyses?		

15	Were appropriate multivariate analysis techniques used?		

16	Did the statistical methods control for confounding and examine subgroups or interactions?		

17	Was there a description on how the final number of participants was established?		

18	Was the (loss to) follow-up of the participants described?		

19	Was the attrition less than 20%?		

20	Was information provided regarding the baseline characteristics of participants?		

21	Was the number of outcome events or summary measures over time reported?		

22	Were the results expressed in an Odds Ratio (OR), Risk Ratio (RR) or Hazard Ratio (HR) with the corresponding 95% confidence interval?		

23	If sub-group analyses were performed, were these clearly described?		

24	Were the key-results described in the discussion?		

25	Were the limitations of the study reported?		

26	Were previous research and the limitations of the study taken into account when an overall interpretation of the study results was provided?		

27	Was the generalisability of the study results described?		

Data regarding design, duration of follow up, sample size, population characteristics, physical frailty indicators, outcome measures and results were extracted from the included studies. The extracted data were not pooled due to the fact that there was a large heterogeneity in the way physical frailty indicators and ADL disability were measured. In order to draw conclusions on the predictive strength of the different indicators, the number of articles reporting a significantly increased risk of ADL disability were counted for each indicator. The number of studies was then split up into studies that only included participants who were free of disability at baseline and studies that included participants not free of disability at baseline. Higher weight was given to studies that only included participants free of disability at baseline (++) compared to studies that included participants with and without disability at baseline (+). Negative weight was given to studies that reported no significant predictive value of the studied indicator (-). In some cases, two different studies that reported positive findings for the same indicator used data from the same cohort. This was taken into account in the interpretation of the results by counting these findings as one.

## Results

### Selection process

**Figure 1:**
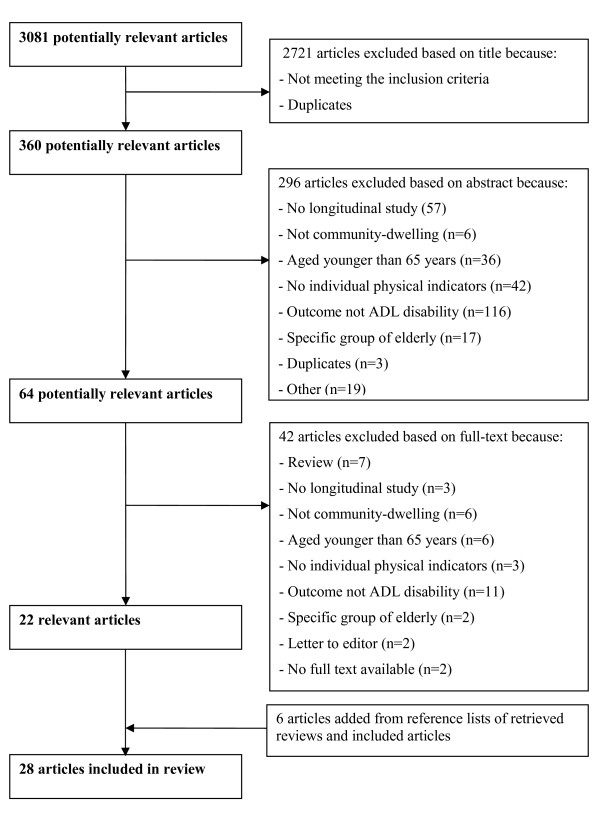
**Flow-chart of selection process**.

The search strategy yielded 3081 potentially relevant articles, after which 360 abstracts were scrutinized, leaving 64 full text publications for final review. After the selection process 28 studies were included in the review (see Figure 1 for details). The agreement between the two reviewers during the selection of abstracts and the selection of full-texts, as measured by Cohen's Kappa, was .74 and .82 respectively which is regarded as substantial to excellent.

### Characteristics of included studies

The characteristics and details of the 28 included studies are presented in Table 2, ordered by year of publication. The main results from the included studies are presented in Table 3. All included studies were longitudinal cohort studies. Various studies reported on the same cohort data: 5 studies were based on the (Hispanic) Established Population for the Epidemiological Study of the Elderly [18-22], 3 studies on the Precipitating Events Project [23-25], 2 studies on the Jerusalem Longitudinal Study [26,27], 3 studies on the Longitudinal Interdisciplinary Study on Aging [28-30], 2 studies on the Cardiovascular Health Study [31,32], 2 studies on the Finland, Italy and The Netherlands Elderly Study [33,34], and 2 studies on the Project Safety cohort [35,36]. The other 9 included studies were based on other cohort studies [37-45]. The duration of follow-up of the studies varied from 1 year to 14 years (mean 5.4, SD 2.9 years). The sample size of the studies varied from 140 to 5727 (mean 1736, SD 2002). 75% of the included studies were published between 1995 and 2005.

**Table 2:** Characteristics of included studies

1^st ^Author (year)	Follow Up	Sample size & Participant characteristics	Physical frailty indicators measured in study*							Quality (0-27)
			W	E	G	M	P	B	O	
Gill et al. (1995) [35]	1 year	563 participants (74% women) with a mean age of 79.1 (SD 4.7)			X			X	X	22

Guralnik et al. (1995) [20]	4 years	1122 men and women aged 71 years and older							X	25

Sonn et al. (1995) [37]	6 years	371 men and women aged 70			X	X				21

Tinetti et al. (1995) [44]	1 year	927 participants (73% women) with a mean age of 79.9 (SD 5.2)			X			X		22

Gill et al. (1996) [36]	3 years	775 participants (74% women) with a mean age of 79.1 (SD 5.0)							X	24

Ostir et al. (1998) [18]	2 years	1365 participants (53% women) with a mean age of 73.3			X			X	X	22

Giampaoli et al. (1999) [33]	4 years	140 men aged 71 to 91				X				20

Wu et al. (1999) [40]	3 years	1321 participants (49% women) 44.4% aged 65-69 and 10.9% aged 80 or older					X			22

Guralnik et al. (2000) [19]	6 years	6534 participants aged 65 years and older			X				X	22

Ishizaki et al. (2000) [30]	3 years	583 participants (56% women) with a mean age of 70.9 (SD 4.9)				X				23

Lee (2000) [38]	7 years	7527 men and women aged 70 years and older					X			20

Sarkisian et al. (2000) [39]	4 years	6632 women with a mean age of 73.0 (4.9)			X	X	X			21

Shinkai et al. (2000) [29]	6 years	736 men and women aged 65 and older			X	X		X		24

Stessman et al. (2002) [27]	7 years	287 participants (51% women) aged 70 years at baseline.					X			20

Wang et al. (2002) [45]	3.4 years	2578 participants (59% women) aged 65 years and older					X			21

Shinkai et al. (2003) [28]	6 years	601 participants (56.1% women) with a mean age of 70.9 (SD 4.9)			X	X		X		22

Al Snih et al. (2004) [22]	7 years	2493 participants (58% women) with a mean age of 72				X				24

Gill et al. (2004) [25]	3 years	754 participants (65% women) aged 70 years or older			X					24

Al Snih et al. (2005) [21]	7 years	1737 Mexican- American participants (58% women) aged 65 and older	X							23

van den Brink et al. (2005) [34]	10 years	560 men aged 70 to 89 years					X			26

Onder et al. (2005) [42]	3 years	884 women (72% white) with a mean age of 78.7 (SD 8.0)			X	X		X	X	23

Jacobs et al. (2008) [26]	7 years	343 men and women aged 70 years.					X			22

Ritchie et al. (2008) [43]	4 years	983 men and women with a mean age of 75.30 (SD 6.72)	X							22

Rosano et al. (2008) [31]	8.4 years	3156 participants (57% women, 71% white) with a mean age of 74.0 (SD 4.6)			X					23

Rothman et al. (2008) [23]	8 years	754 men and women with a mean age of 78.4 (SD 5.3)	X	X	X	X	X			22

Gill et al. (2009) [24]	9 years	722 participants (65.2% women) with a mean age of 78.4 (SD 5.2)				X			X	25

Arnold et al. (2010) [32]	14 years	3278 participants (61% women, 83% white) with a mean age of 80	X							23

Balzi et al. (2010) [41]	3 years	897 Italian men and women, aged 65 to 102					X		X	21

**Total number of studies per indicator**			**4**	**1**	**12**	**10**	**9**	**6**	**8**	

* W: Weight loss, E: Exhaustion, G: Gait speed, M: Muscle strength, P: Physical activity, B: Balance, and O: Other indicators.

**Table 3:** Main results of included studies

1^st ^Author (year)	Study Results
Gill et al. (1995) [35]	Each performance test (chair stand, rapid gait, 360° turn, bending over, foot taps, and hand signature) is significantly associated with the onset of functional dependence in ADL disability. Adjusted Risk Ratios (RR) vary from 1.2 (.7-2.0) for foot taps to 2.4 (1.4-4.2) for rapid gait.

Guralnik et al. (1995) [20]	Elderly people with lowest lower extremity function have a higher risk of ADL disability compared to elderly people in higher lower extremity function groups. RR 4.2 (2.3-7.7). Elderly people in the moderate group have a higher risk of ADL disability compared to elderly people in the high group. RR 1.6 (1.0-2.6).

Sonn et al. (1995) [37]	Walking speed and grip strength at age 70 are significantly associated with incident ADL disability at age 76.

Tinetti et al. (1995) [44]	Elderly people with lower usual gait speed, lower rapid gait speed, or lower balance have a higher risk of functional dependence in ADL. OR 2.0 (1.5-2.7), 2.3 (1.7-3.2), and 2.0 (1.5-2.7) respectively.

Gill et al. (1996) [36]	Elderly people in the lowest quartile of physical function (measured by walking, turning, chair stands) have a higher risk of functional dependence in ADL. RR 2.1 (1.4-3.0).

Ostir et al. (1998) [18]	Elderly people in the lowest quartile of walking speed, balance, and chair stands have a higher risk of ADL disability after a 2-year follow-up compared to elderly people in the highest quartile. OR 5.4 (1.2-23.6), OR 2.4 (1.0-5.4), and OR 2.8 (1.2-6.4) respectively.

Giampaoli et al. (1999) [33]	Elderly men with higher hand grip strength have a lower risk of disability compared to men with lower hand grip strength. OR .97 (.96-.99).

Wu et al. (1999) [40]	Elderly people who participated regularly in exercise had a lower risk of becoming chronically ADL disabled after a 3-year follow-up. RR .52 (.39-.68).

Guralnik et al. (2000) [19]	Elderly people with low lower extremity function have a higher risk of ADL disability compared to elderly people with high lower extremity function. RR ranging from 3.4 (1.7-7.1) to 7.4 (1.8-30.5). Elderly people with moderate lower extremity function have a higher risk of ADL disability compared to elderly people with high lower extremity function. RR ranging from 1.2 (.7-2.2) to 2.0 (.7-5.3). Gait speed alone performed almost as well as total lower extremity function in predicting incident disability.

Ishizaki et al. (2000) [30]	Elderly people with higher hand grip strength (1kg) have a lower risk of developing disability in basic ADL within the next 3 years. OR .91 (.84-.97).

Lee (2000) [38]	Elderly people who think that they are less active than other people their age have a higher risk of ADL disability compared to people who think that they are a lot more active than other people their age. OR 1.65 (1.14-2.39).

Sarkisian et al. (2000) [39]	Elderly people in the lowest quintile of gait speed have a higher risk of decline in basic ADL. OR 2.29 (1.66-3.17). Elderly people in the lowest quintile of exercise level also have a higher risk of basic ADL decline. OR 1.47 (1.06-2.05).

Shinkai et al. (2000) [29]	Maximum walking speed, usual walking speed, balance, and grip strength are significant predictors of the onset of functional ADL dependence after a 6-year follow-up in elderly people who are aged 65-74 and 75 or older. For elderly people in the lowest quartile the HR ranged from 2.21 (1.23-3.97) to 6.18 (3.16-12.1).

Stessman et al. (2002) [27]	Elderly people who are not physically active or who do not exercise at least four days a week at age 70 have a higher risk of ADL disability after a 7-year follow- up compared to elderly people who are physically active at age 70. OR for men 4.3 (1.1-17.1), OR for women 8.5 (2.0-36.2).

Wang et al. (2002) [45]	Elderly persons who exercise regularly have a decreased age-adjusted risk of functional decline in ADL.

Shinkai et al. (2003) [28]	Elderly people in the lowest quartile of hand grip strength, balance, usual walking speed or maximal walking speed have a higher risk of disability in basic ADL. HR 1.22 (1.07-1.39), 1.41 (1.22-1.62), 1.31 (1.14-1.50), and 1.40 (1.22-1.61) respectively.

Al Snih et al. (2004) [22]	Men and women in the lowest quartile of hand grip strength have a higher risk of ADL limitations in the next 7 years. HR for men 1.9 (1.14-3.17) and HR for women 2.28 (1.59-3.27).

Gill et al. (2004) [25]	Slow gait speed is associated significantly with the development of insidious disability. OR 2.4 (1.4-4.1).

Al Snih et al. (2005) [21]	Elderly people with weight loss of 5% or more within a 2-year follow-up after baseline have a higher risk of lower body ADL disability compared to elderly people with stable weight. Adjusted OR 1.43 (1.06-1.95).

van den Brink et al. (2005) [34]	Compared to the lowest tertile of total physical activity men from the middle and highest tertile have a lower risk of disability. OR .56 (.32-.99) and OR .50 (.29- .88) respectively.

Onder et al. (2005) [42]	Balance, chair stands, and walking speed were significant predictors of progressive incident ADL disability. Walking speed was also a significant predictor of catastrophic incident disability.

Jacobs et al. (2008) [26]	Elderly people who go out less then daily at age 70 have a higher risk of incident dependence in ADL compared to elderly people who go out daily at age 70. RR 6.9 (1.4-34.0).

Ritchie et al. (2008) [43]	A history of unintentional weight loss at baseline predicts more rapid decline in ADL.

Rosano et al. (2008) [31]	Gait speed is a significant predictor of disability. HR .88 (.80-.96). This HR remains when controlling for age, sex, race, education, and possible confounders.

Rothman et al. (2008) [23]	Slow gait speed, low physical activity and weight loss are significant predictors of chronic incident disability. HR 3.0 (2.3-3.8), HR 2.1 (1.7-2.6), and HR 1.7 (1.4-2.1) respectively. Exhaustion and grip strength do not predict chronic incident disability

Gill et al. (2009) [24]	Poor grip strength was associated with 3 subtypes of disability. OR ranging from 1.42 (1.03-1.95) to 1.80 (1.04-3.12). Lower extremity performance score was significantly associated with 5 subtypes of ADL disability. OR ranging from 1.10 (1.04-1.17) to 1.35 (1.24-1.47).

Arnold et al. (2010) [32]	Elderly people with weight loss of 5% or more between consecutive annual visits have a higher risk of incident ADL disability compared to elderly people with stable weight. Adjusted OR 1.27 (1.10-1.46).

Balzi et al. (2010) [41]	High level of physical activity compared to sedentary state is associated with a lower incidence of ADL disability after a 3-year follow-up. OR .30 (.12-.76). Lower extremity performance score is a significant predictor of disability.

The quality of the 28 included studies varied between 20 and 26 (27 was highest score possible). The mean quality score was high: 22.5 (SD 1.6) points. For each quality item, the Cohen's Kappa was calculated to measure the agreement between the two reviewers. The Kappas varied between 1.00 and .13. Agreement was high (Kappa > .70) for 18 items, moderate (Kappa between .40 and .70) for 7 items, and low (Kappa < .40) for 2 items. Of the included studies, 50% had a follow-up of 5 years or longer [19,21-24,26-29,31,32,34,37,38] and 68% included only participants who were free from disability at baseline [18-23,25,26,28-32,34-36,40-42]. Only 11% of the included studies did not use a standardized or valid measurement to measure the physical frailty indicators [26,27,30] and only 4% did not use a standardized or valid measurement to measure ADL disability [38]. All studies used appropriate multivariate analysis and corrected for confounders in their analyses. 39% of the included studies had an attrition below 20% [19,20,24,25,29,34-36,40,42,44].

A variety of physical frailty indicators was measured in the included studies: weight loss, exhaustion, gait speed/walking speed/gait, muscle strength/grip strength, physical activity, balance, lower extremity function, chair stand, 360° turn, bending over, foot taps, and hand signature. There was considerable variation in the way the same indicators were measured and operationalized in different studies. Also, different cut-off points were used in different studies. More detailed information regarding the measurement of the indicators is presented in Additional file 1.

The operationalization of ADL disability also varied across studies. Some studies defined disability as dependency in ADL at follow-up, others as difficulty in ADL at follow-up, and some studies used chronic ADL disability as an outcome measure. Some studies only measured disability in 4 different ADL, whereas others measured disability in 5, 6, or 7 ADL. More detailed information regarding the measurement of ADL disability is also presented in Additional file 1.

### Predictive value of physical frailty indicators on ADL disability

For each individual physical frailty indicator the evidence regarding the predictive value is described below. The information is summarized in Table 4.

**Table 4:** Predictive strength of physical frailty indicators on ADL disability

Physical frailty indicator	Total number of studies	Number of studies, only including participants free of disability at baseline, that reported a significant increased risk of ADL disability (Number of cohorts) ++	Number of studies, including both participants free and not free of ADL disability at baseline, that reported a significant increased risk of ADL disability (Number of cohorts) +	Number of studies reporting no significant increased risk of ADL disability (Number of cohorts) -
Weight loss	4	4 (4)	0 (0)	0 (0)

Exhaustion	1	0 (0)	0 (0)	1 (1)

Gait speed	12	9 (6)	3 (3)	0 (0)

Muscle strength	10	4 (2)	3 (3)	3 (3)

Physical activity	9	5 (5)	4 (4)	0 (0)

Balance	6	4 (3)	1 (1)	1 (1)

Others:				
- Lower extremity function	5	4 (4)	1 (1)	0 (0)
- Chair stands	3	2 (2)	1 (1)	0 (0)
- 360° turn, bending over, foot taps, hand signature	1	1 (1)	0 (0)	0 (0)

#### Weight loss

Four studies provided information regarding the predictive value of weight loss on ADL disability. These four studies were based on separate cohorts that only included participants who were free of disability at baseline [21,23,32,43]. All four studies concluded that elderly people who report (unintentional) weight loss have a significant higher risk to develop ADL disability.

#### Exhaustion

Only one study reported on the predictive value of exhaustion on ADL disability [23]. This study concluded that feelings of exhaustion are not a significant predictor of ADL disability in elderly people.

#### Gait speed

Twelve studies provided information about the predictive value of gait speed (walking speed) as an individual physical frailty indicator on ADL disability [18,19,23,25,28-30,35,37,39,42,44]. All studies concluded that elderly people with slower gait speed have a higher risk of developing ADL disability. Nine studies were based on six separate cohort studies that only included participants free of ADL disability at baseline [18,19,23,25,28,29,31,35,42]. The other three studies were separate cohort studies that included participants with and without disability at baseline [37,39,44].

#### Muscle strength

Ten studies provided information about the predictive value of muscle strength or hand grip strength on ADL disability [22-24,28-30,33,37,39,42]. Seven studies concluded that grip strength is a significant predictor of ADL disability [22,24,28-30,33,37]. Four studies, using data from two separate cohorts, only included participants free of ADL disability at baseline [22,28-30]. The other three separate cohort studies with a positive finding included participants with and without ADL disability at baseline [24,33,37]. Three studies concluded that grip strength is not a significant predictor of ADL disability [23,39,42].

#### Physical activity

Nine studies reported on the predictive value of physical activity or exercise on ADL disability [23,26,27,34,38-41,45]. All nine studies concluded that elderly people who are more physically active or who participate in exercise more regularly have a lower risk of developing ADL disability. Five out of these nine studies only included participants free of ADL disability at baseline [23,26,34,40,41]. These five studies were based on five separate cohort studies. The other four separate cohort studies included participants with and without disability at baseline [27,38,39,45].

#### Balance

Six studies provided information about the predictive value of balance [18,28,29,35,42,44]. Five out of these six studies concluded that elderly people with poorer balance have a higher risk of developing ADL disability [28,29,35,42,44]. These five studies were based on three separate cohorts that only included participants free of ADL disability at baseline [28,29,35,42]. The other study with a positive finding included participants with and without ADL disability at baseline [44].

#### Other physical frailty indicators

Eight studies reported on the predictive value of physical frailty indicators that were not mentioned above namely: lower extremity function, chair stands, 360° turn, bending over, foot taps, and hand signature.

Five of these studies reported on lower extremity function [19,20,24,36,41]. In all five studies, lower extremity function appeared to be a significant predictor of ADL disability. Elderly people with low lower extremity function had a higher risk of ADL disability at follow-up compared to people with moderate or high lower extremity function. Four of these studies were based on four separate cohorts that only included participants who were free of disability at baseline [19,20,24,36]. The other cohort study included participants with and without ADL disability at baseline [41].

Three studies investigated the predictive value of chair stands on ADL disability and concluded that this indicator is a significant predictor of ADL disability [18,35,42]. Two studies were based on two separate cohorts that only included participants who were free of disability at baseline [18,35]. The other cohort study included participants with and without ADL disability at baseline [42].

The study by Gill et al. [35] also investigated the predictive value of 360° turn, bending over, foot taps, hand signature and concluded that all indicators were predictors for ADL disability. The cohort study only included participants who were free of disability at baseline.

## Discussion

This review provides evidence that physical frailty indicators are predictors of ADL disability in community-dwelling elderly people aged 65 years and older. Elderly people with unintended weight loss, slower gait speed, lower grip strength, lower physical activity, lower exercise, poor balance, or low lower extremity function have a higher risk of ADL disability in the future. Apparently, physical frailty indicators do not only predict disability when they are related in a frailty phenotype [12] but also independent of each other.

The number of studies that focused on the predictive value on ADL disability differed per physical frailty indicator. Almost half of the included studies investigated the predictive value of gait speed whereas only one study reported on exhaustion. Besides that, there were large variations in the measurement of frailty indicators and ADL disability across the 28 included studies. Therefore, it is difficult to draw firm conclusions regarding the predictive power of the different indicators compared to each other. Nevertheless, taking into account the number of studies per indicator that suggested a significantly increased risk of ADL disability for this indicator provides some insight into the predictive value. Slow gait speed and low physical activity or exercise seem to have the highest predictive power, followed by weight loss, lower extremity function, balance, muscle strength, and other indicators. These findings should be interpreted with caution because pooling of the data from different studies was not possible.

The follow-up period of the cohorts varied across the included studies. Three studies had a follow-up of 1 or 2 years, six studies had a follow-up of 3 years, and the rest of the studies had a follow-up longer than 3 years. From this can be concluded that certain indicators predict disability in the short-term, long-term or both. For example, gait speed and balance predict the development of ADL disability after a follow up of one year [35,44] and 6 years [29] and physical activity predicts the development of disability after a follow-up of 3 years [40,41] and 10 years [34]. For the identification of elderly people who could benefit from an intervention that prevents ADL disability, it is more useful to know the 'short-term' predictive value of the physical frailty indicators. It makes more sense to start with a preventive intervention when 'short-term predictors' are present in elderly people compared to a situation in which it will take another 6 years (or longer) before disability will develop.

A large part of the included studies had a relatively long follow-up period. It would be interesting to see whether indicators that predict disability after a long period of time, are also predictors of disability on the short term, e.g. 1 year. Besides that, it would also be useful to know how much the functioning of the physical frailty indicators would have to decrease before disability starts to develop in elderly people. Many of the included studies used quartile or quintile scores to define high or low physical functioning in the frailty indicators. As a result, many of these studies reported limited generalisability of their findings. Clear cutoff points have not been established yet for all indicators. This could be a focus of future research and should also be taken into account when developing interventions that can prevent disability in community-dwelling elderly people.

The only physical frailty indicator that appeared not to predict ADL disability was exhaustion. However, only one study included in this review focused on this [23]. Exhaustion is a feeling not only related to physical functioning but also to mental/psychological functioning. Since the search strategy focused strongly on physical functioning, some studies regarding exhaustion might not have been retrieved. Another possibility might be that hardly any studies focusing on the predictive value of exhaustion have been conducted.

### Limitations of the review

Despite the effort of the authors to conduct a sensitive search strategy, some relevant studies or unpublished articles may not have been retrieved. It is also very remarkable that almost all selected studies showed positive results and were of (very) high quality. This may indicate publication bias.

A remark must be made regarding the quality scores of the included studies which were quite high. This is not necessarily a limitation of the study but rather an exceptional finding. The high quality scores might have been caused by the selection criteria which allowed only prospective cohort studies to be included. Another possible explanation could be that the criteria that were used to assess the quality of the studies did not only refer to the methodological quality but also to the quality of reporting. This might have elevated the quality scores compared to when the quality of reporting criteria would not have been taken into account.

The term frailty was first introduced in the 1980's. If earlier studies used different definitions or measurement methods for frailty or its adverse outcomes compared to more recent studies, this might have introduced the possibility of time-lapse bias. However, the probability of this type of bias is probably small due to the broad search terms that were used in the search strategy.

Many studies that were included in the review were based on secondary data-analyses. If measurement of the indicators or ADL disability was not the primary aim of the study, this might have resulted in the use of suboptimal measurement methods. However, the quality assessment of the included articles revealed that the majority of the studies used standardized or validated measurements for the indicators and outcome variables.

## Conclusions

This review showed that physical frailty indicators predict ADL disability in community-dwelling elderly people. Slow gait speed and low physical activity/exercise seem to be the most powerful predictors followed by weight loss, lower extremity function, balance, muscle strength, and other indicators. Monitoring these indicators might be useful for identifying elderly people who could benefit from an intervention aimed at preventing ADL disability. Such an intervention could partly relieve the burden that frailty places on individuals, care providers and the health care system as a whole.

## Competing interests

The authors declare that they have no competing interests.

## Authors' contributions

JV contributed to the development of the search strategy, conducted the search, analyzed the articles (1^st ^reviewer), and drafted the manuscript. JCLN contributed to the development of the search strategy, analyzed the articles (2^nd ^reviewer), and helped to draft the manuscript. EvR contributed to the development of the search strategy and helped to draft the manuscript. MDS contributed to the development of the search strategy, was consulted during the in- and exclusion of articles (3^rd ^reviewer), and helped to draft the manuscript. LPdW was the project supervisor and contributed to conceptualization and development of the search strategy and helped to draft the manuscript.

All authors read and approved the final manuscript.

## Pre-publication history

The pre-publication history for this paper can be accessed here:

http://www.biomedcentral.com/1471-2318/11/33/prepub

## Supplementary Material

Additional file 1 **Measurement of physical frailty indicators and ADL disability**. The table in Additional file 1 shows how physical frailty indicators and ADL disability were measured in the 28 included articles. Click here for file
