# Supplementary material for: Predicting ADL disability in community-dwelling elderly people using physical frailty indicators: a systematic review
Source: BMC Geriatr. 2011 Jul 1;11:33. doi: 10.1186/1471-2318-11-33 (PMC3142492; doi:10.1186/1471-2318-11-33)
Supplement: Additional file 1 — Measurement of physical frailty indicators and ADL disability. The table in Additional file 1 shows how physical frailty indicators and ADL disability were measured in the 28 included articles. [file 1471-2318-11-33-S1.PDF]

Additional file 1 – Measurement of physical frailty indicators and ADL disability

Article	Measurement Indicator	Measurement Disability
	Weight loss	
Al Snih et al. (2005) [21]	Weight was measured at baseline and after 2 years follow-up. Weight loss was defined as losing 5% or more of the total body weight after 2 years follow-up.	Participants were asked whether they needed help in four ADL (bathing, dressing, transferring, eating). Lower body ADL limitation was defined as needing help with or being unable to perform one or more ADL.
Ritchie et al. (2008) [43]	Participants were asked at baseline whether they lost more than 10 pounds in the past year and whether this was intentional or not. Answers were coded into three categories: no loss, intentional loss, and unintentional loss.	Self-reported difficulty with six ADL (eating, toileting, dressing, transferring, bathing, and walking) was measured on a 4-point scale (0-3). Sum scores were calculated to indicate the level of disability.
Rothman et al. (2008) [23]	Participants were asked at baseline whether they lost more than 10 pounds in the past year. Answers were coded into two categories: yes and no.	Chronic disability was defined as a new ADL disability that was present for at least 3 consecutive months (bathing, dressing, walking and transferring).
Arnold et al. (2010) [32]	Weight was measured during annual exams. Weight loss was defined as a loss of 5% of total body weight between consecutive annual visits or from baseline, without an intervening 5% gain	Disability was defined as any difficulty with one or more of six ADL (walking around the home, dressing, eating, bathing, toileting, and getting out of a bed or chair).
	Exhaustion	
Rothman et al. (2008) [23]	Participants were asked how often 'they felt like everything they did was an effort' and how often 'they could not get going'. Exhaustion was defined as answering 'much or most of the time' to one of these questions.	Chronic disability was defined as a new ADL disability that was present for at least 3 consecutive months (bathing, dressing, walking and transferring).
	Gait speed	
Gill et al. (1995) [35]	Rapid gait speed was measured while participants walked back and forth over a 10-foot course as quickly as they could. Slow gait speed was defined as the lowest quartile.	Disability was defined as receiving personal assistance or being completely dependent in one or more ADL (bathing, dressing, transferring, eating, personal grooming, and walking across a small room).
Sonn et al. (1995) [37]	Maximal walking speed was measured over 30 meters indoors.	Disability was defined by dependency on others in one or more of five personal ADL (bathing, toileting, transferring, feeding, dressing).

Tinetti et al. (1995) [44]	Participants were asked to walk 10 foot back and forth as fast as they could. Slow gait speed was defined as the lowest quartile.	Disability was defined as self-reported need for human help with one or more ADL (eating, grooming, bathing, dressing, transferring, walking around the house).
Ostir et al. (1998) [18]	Participants were asked to walk 8 foot twice at a usual pace. The fastest timed walk was used for scoring purposes. Slowest gait speed was defined as taking longer than 9 seconds to walk 8 foot (lowest quartile).	Disability was defined as ADL dependency (activities not specified in article).
Guralnik et al. (2000) [19]	Participants were asked to walk 8 foot twice at a usual pace. The fastest timed walk was used for scoring purposes. Slowest gait speed was defined as taking longer than 5.7 seconds to walk 8 foot (lowest quartile).	Disability was defined as inability to perform one or more ADL (transferring, toileting, bathing, walking across a small room) without help from another person.
Sarkisian et al. (2000) [39]	Gait speed was determined by measuring the time in seconds needed to walk 6 meters at a rapid pace. Slow gait speed was defined as the lowest quintile.	Disability was defined as a decrease of one or more ADL able to complete without assistance between visits 2 and 4.
Shinkai et al. (2000) [29]	Usual and maximum walking speed (measured over 5 meter of an 11 meter course)	Dependence in ADL was defined as needing help from someone else or being unable to perform one of five ADL (bathing, dressing, walking, eating and continence).
Shinkai et al. (2003) [28]	Walking speed was measured over a 5 meter distance (between the 3 and 8 meter marks from the start of the walkway). For maximal walking speed, the fastest result of two attempts was used. Slow gait speed was defined as the lowest quartile.	Disability was defined as needing help from someone else or being unable to perform one or more ADL (bathing, dressing, walking, eating and continence).
Gill et al. (2004) [25]	Participants walked back and forth over a 3 meter course as quickly as possible. Slow gait speed was defined as a score of 10 seconds or slower.	Disability was defined as needing help from someone else or being unable to perform one or more ADL (bathing, dressing, walking around at home, transferring).
Onder et al. (2005) [42]	Participants were asked to walk over a 4 meter course twice, as fast as possible. Slow walking speed was defined as walking slower than 0.09 m/second or being unable to complete the test.	Participants were classified with progressive disability if they reported a lot of difficulty or inability to perform an ADL between follow-ups 2 and 6 and they had a little or some difficulty at one or both of the semiannual assessments before the onset of disability (bathing, dressing, eating, transferring, toileting).
Rosano et al. (2008) [31]	Participants were asked to walk 15 foot at a usual pace. Slow gait speed was defined as walking slower than 1 m/second.	Incident disability was defined as self-reported difficulty or inability to perform at least one of six ADL without assistance (bathing, dressing, eating, toileting, walking around the home, and transferring).
Rothman et al.	The slow gait speed criterion was met if the participant took more	Chronic disability was defined as a new ADL disability that was

(2008) [23]	time than 10 seconds to walk back and forth over a 10-foot course as quickly as possible.	present for at least 3 consecutive months (bathing, dressing, walking and transferring).
	Muscle strength	
Sonn et al. (1995) [37]	Grip stenght in left and right hand was measured with a Vigorimeter at an elbow-angle of 90° and with the shoulder joint in neutral position (medium-sized ball for women and big ball for men). The strongest measure was scored.	Disability was defined by dependency on others in one or more of five personal ADL (bathing, toileting, transferring, feeding, dressing).
Giampaoli et al. (1999) [33]	Upper extremity strength was assessed by the hand-grip test using the Martin dynamometer, women and men were divided into quartiles.	Disability was defined by inability to perform one or more ADL without help (walking around in the house, transferring, toileting, bathing, dressing).
Ishizaki et al. (2000) [30]	Handgrip strength was measured twice in the dominant had using a hand dynamometer. The best out of these two efforts was used in the analyses.	Disability was defined as a loss of independence in one or more ADL (walking, feeding, continence, bathing, dressing).
Sarkisian et al. (2000) [39]	Maximum grip strength was measured with a grip dynamometer in both hands and averaged. Low grip strength was defined as the lowest quintile.	Disability was defined as a decrease of one or more ADL able to complete without assistance between visits 2 and 4.
Shinkai et al. (2000) [29]	Handgrip strength was evaluated twice using a mechanical dynamometer in the dominant hand. The strongest out of two efforts was used in the analyses. Low grip strength was defined by the lowest quartile.	Dependence in ADL was defined as the subject needing help from someone else or being unable to perform one of five ADL (bathing, dressing, walking, eating and continence).
Shinkai et al. (2003) [28]	Handgrip strength was evaluated twice using a mechanical dynamometer in the dominant hand. The strongest out of two efforts was used in the analyses. Low grip strength was defined by the lowest quartile.	Disability was defined as needing help from someone else or being unable to perform one or more ADL (bathing, dressing, walking, eating and continence).
Al Snih et al. (2004) [22]	Hand grip strength in the dominant hand was measured in kilograms using a hand-held dynamometer in a sitting position with elbow residing on the table.	Disability was defined by needing help or being unable to perform one or more ADL (walking across a small room, bathing, grooming, dressing, eating, transferring, toileting).
Onder et al. (2005 [42]	The best effort of three measurements of grip strength was used in the analyses. Participants who scored 5 kg or less were classified as worst performers.	Participants were classified with progressive disability if they reported a lot of difficulty or inability to perform an ADL (bathing, dressing, eating, transferring, toileting) between follow-ups 2 and 6 and they had a little or some difficulty at one or both of the semiannual assessments before the onset of disability.
Rothman et al.	Grip strength was measured using a handheld dynamometer and	Chronic disability was defined as a new ADL disability that was

(2008) [23]	considered low when below sex- and body mass index-specific cutoff points [11].	present for at least 3 consecutive months (bathing, dressing, walking and transferring).
Gill et al. (2009) [24]	Grip strength was measured using a handheld dynamometer and considered low when below sex- and body mass index-specific cutoff points [11].	Disability is defined as needing help with one or more ADL. Five different types of disability were defined (transient, short-term, long-term, recurrent, unstable).
	Physical activity	
Wu et al. (1999) [40]	Participants who exercised (folk dancing, hiking, jogging or walking) at least twice a week were considered as routine recreational exercisers.	Participants incapable of performing one of six ADL (bathing, dressing, transfers, toileting, eating, walking inside the home) independently for more than 3 months were considered chronically disabled.
Lee (2000) [38]	Participants were asked whether they think they are more, the same, or less active compared to others their age to assess the level of physical activity. Besides that, participants who thought they get enough exercise were defined as exercisers.	Disability was defined as having difficulty with or being dependent in ADL (bathing, dressing, eating, transferring, toileting)
Sarkisian et al. (2000) [39]	Exercise level was examined with a modified Paffenbarger survey. Participant with scores in the lowest quintile were classified as low activity level.	Disability was defined as a decrease of one or more ADL able to complete without assistance between visits 2 and 4.
Stessman et al. (2002) [27]	Participants who exercised at least four days a week were classified as exercisers.	Disability was defined as independency in four ADL (bathing, toileting, dressing, rising from a chair).
Wang et al. (2002) [45]	Participants who participated in one of the following forms of exercise at least three times per week were classified as performing regular exercise (walking, hiking, bicycling, aerobics, swimming, water aerobics, weight training or other exercise).	The level of disability in six ADL (walking around the house, bathing, dressing, transferring, eating, toileting) was assessed on an scale from 0 to 3 resulting in a maximum score.
van den Brink et al. (2005) [34]	Physical activity was measured with a standardized self-administered questionnaire that measures duration and intensity. Participants were divided into three categories: low, middle, high activity.	Disability was defined as needing help with one or more ADL (walking, transferring, eating, washing, dressing, toileting).
Jacobs et al. (2008) [26]	Physical activity was measured by asking participant how often they go outdoors (daily, nearly daily, 2-3 times a week, once a week, less than once a week)	Disability was defined as needing assistance of another person in one or more ADL (transferring, dressing, bathing, toileting, eating, continence).
Rothman et al. (2008) [23]	Physical activity was measured with the PASE questionnaire. Low physical activity was defined as a score below 64 for men and below 52 for women.	Chronic disability was defined as a new ADL disability that was present for at least 3 consecutive months (bathing, dressing, walking and transferring).

Balzi et al. (2009) [41]	Physical activity was measured with an ordinal 7-point scale and dichotomized into absent/light activity vs. moderate activity.	Worsening and development of new disability was defined as needing help from another person in ADL.
	Balance	
Gill et al. (1995) [35]	Balance was examined as part of the balance subscale of the Performance Oriented Mobility Assessment.	Disability was defined as receiving personal assistance or being completely dependent in one or more ADL (bathing, dressing, transferring, eating, personal grooming, and walking across a small room).
Tinetti et al. (1995) [44]	Participants performed various balance maneuvers from the Performance Oriented Mobility Assessment including side-by-side, sterna nudge, tandem, and one-leg stands	Disability was defined as self-reported need for human help with one or more ADL (eating, grooming, bathing, dressing, transferring, walking around the house).
Ostir et al. (1998) [18]	Participants performed three balance related tasks of increasing difficulty (side-by-side, semi-tandem and tandem stand) which were timed. Performance was scored on a 3-point scale.	Disability was defined as ADL dependency (activities not specified in article).
Shinkai et al. (2000) [29]	Balance was measured by timing how long participants could stand on 1 leg until balance was lost. The scores were divided into quartiles.	Dependence in ADL was defined as needing help from someone else or being unable to perform one of five ADL (bathing, dressing, walking, eating and continence).
Shinkai et al. (2003) [28]	Balance was measured by timing how long participants could stand on 1 leg until balance was lost. The scores were divided into quartiles.	Disability was defined as needing help from someone else or being unable to perform one or more ADL (bathing, dressing, walking, eating and continence).
Onder et al. (2005) [42]	Participants performed a chair stand test and balance test.	Participants were classified with progressive disability if they reported a lot of difficulty or inability to perform an ADL between follow-ups 2 and 6 and they had a little or some difficulty at one or both of the semiannual assessments before the onset of disability (bathing, dressing, eating, transferring, toileting).
	Others	
Gill et al. (1995) [35]	The following timed performance tests were administered to the participants: ten foot taps, three chair stands, 360° turn, time to bend over and pick up a pen, and time to pick up a pencil and complete a signature. Participants performed these tests as fast as possible. Participants were divided into quartiles of performance.	Disability was defined as receiving personal assistance or being completely dependent in one or more ADL (bathing, dressing, transferring, eating, personal grooming, walking across a small room).
Guralnik et al. (1995) [20]	A summary performance score was created for each participant by adding the scores for the tests of standing balance, walking, and	Disability was defined as the inability to perform one or more of the basic activities without the help of another person (moving from a

	repeatedly rising from a chair. Scores ranged from 3 to 12 and three groups were created based on this score. These groups were compared in the analysis.	bed to a chair, using the toilet, bathing, walking across a small room).
Gill et al. (1996) [36]	The following timed performance tests were administered to the participants: three chair stands, 360° turn, and rapid gait back and forth over a 10-foot course. Participants performed these tests as fast as possible. Participants were divided into quartiles of performance.	Disability was defined as requiring personal assistance in one or more of seven ADL at either the 1- or 3-year follow-up interview (walking around the house, bathing, dressing, transferring, eating, toileting, personal grooming).
Ostir et al. (1998) [18]	For each participant, an overall lower body function score was calculated by summing the three individual scores on an 8-foot walk test, a chair stands test, and a balance test. Total summed scores ranged from a low of 1 to a high of 11. Participants were divided into quartiles of performance.	Disability was defined as ADL dependency (activities not specified in article).
Guralnik et al. (2000) [19]	Lower extremity function was measured using tests of gait speed, standing balance, and time to rise from a chair five times. Scores ranged from 3 to 12 and three groups were created based on this score. These groups were compared in the analysis.	Disability was defined as inability to perform one or more ADL without help from another person (transferring, toileting, bathing, walking across a small room).
Onder et al. (2005) [42]	Summary performance scores for lower extremities were calculated by adding the rescaled scores for the walking speed test, chair stands test, and standing balance test.	Participants were classified with progressive disability if they reported a lot of difficulty or inability to perform an ADL between follow-ups 2 and 6 and they had a little or some difficulty at one or both of the semiannual assessments before the onset of disability (bathing, dressing, eating, transferring, toileting).
Gill et al. (2009) [24]	Three tests were administered to all participants to test physical performance: standard balance maneuvers, three timed chair stands, and timed rapid gait. The scores on these tests were added creating a performance score between 0 and 12 for each participant.	Disability is defined as needing help with one or more ADL. Five different types of disability were defined (transient, short-term, long-term, recurrent, unstable).
Balzi et al (2010) [41]	Walking speed over 4 meters, five timed repeated chair rises and standing balance were measured in all participants. The scores on these tests were added creating a performance score between 0 and 12 for each participant. Three groups were created based on this score. These groups were compared in the analysis.	Worsening and development of new disability was defined as needing help from another person in ADL.
